# Supplementary material for: Complex‐centric proteome profiling by SEC‐SWATH‐MS
Source: Mol Syst Biol. 2019 Jan 14;15(1):e8438. doi: 10.15252/msb.20188438 (PMC6346213; doi:10.15252/msb.20188438)
Supplement: Supplementary file 6 — Dataset EV5 [file MSB-15-e8438-s006.zip › feature_plots_corum/3082.pdf]

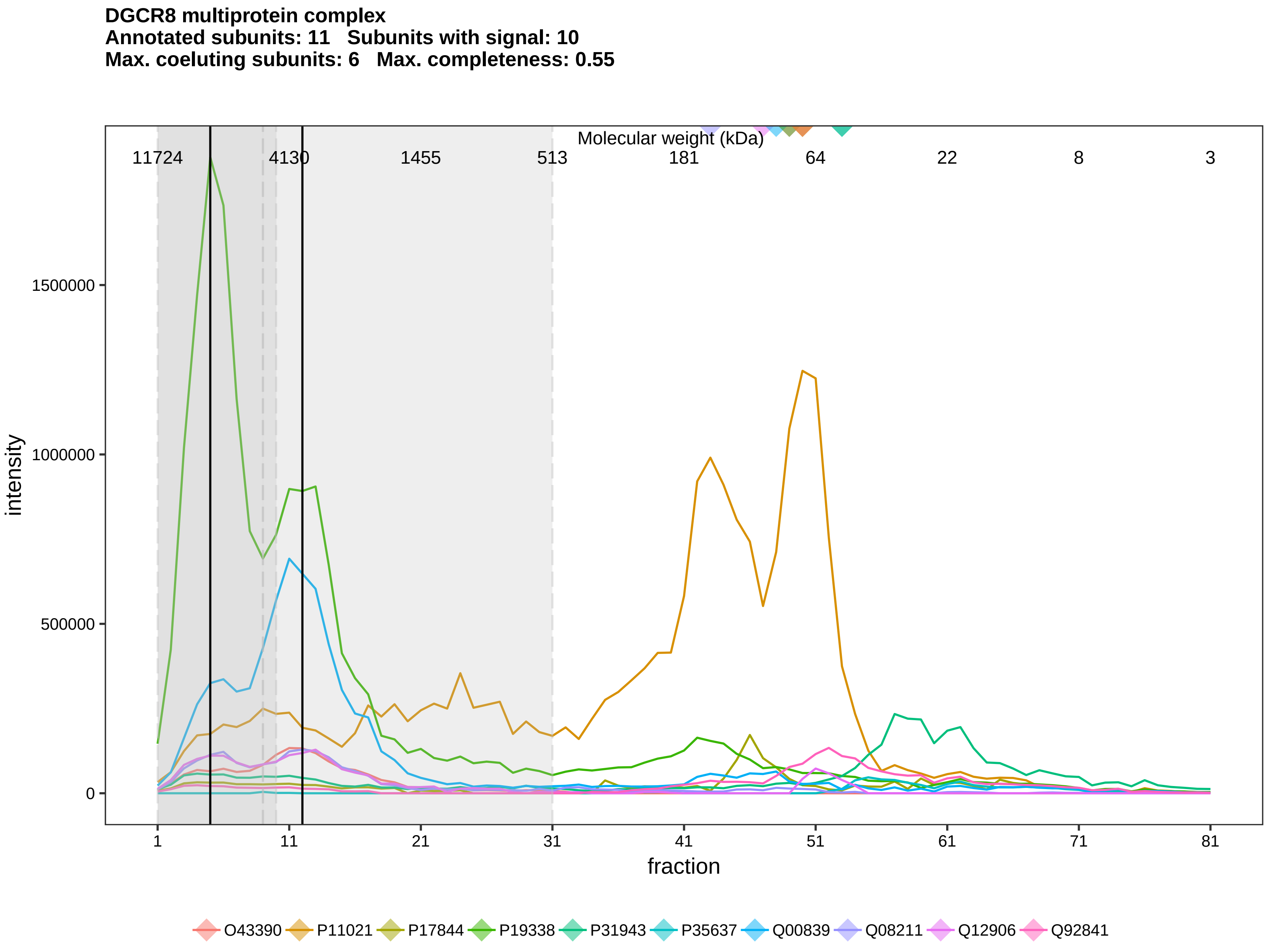

DGCR8 multiprotein complex
Annotated subunits: 11 Subunits with signal: 10
Max. coeluting subunits: 6 Max. completeness: 0.55
Molecular weight (kDa)
11724
4130
1455
513
181
64
22
8
3
intensity
1500000
1000000
500000
0
1
11
21
31
41
51
61
71
81
fraction
O43390
P11021
P17844
P19338
P31943
P35637
Q00839
Q08211
Q12906
Q92841
